# Spontaneous Cerebrospinal Fluid Rhinorrhea in Pre-operative Pituitary Adenoma: A Report of Two Cases

**DOI:** 10.7759/cureus.71642

**Published:** 2024-10-16

**Authors:** Jessica E Eisold, Dace Dimante, Jonathan Pollock, Alireza Shoakazemi, Nemanja Stojanovic

**Affiliations:** 1 Medicine, American University of the Caribbean School of Medicine, Cupecoy, SXM; 2 Neurosurgery, Barking, Havering And Redbridge University Hospitals National Health Services (NHS) Trust, Romford, GBR; 3 Neurosurgery, Queen's Hospital, Romford, GBR; 4 Endocrinology and Diabetes, Queen's Hospital, Romford, GBR

**Keywords:** acth secreting tumor, cerebrospinal fluid fistula, endonasal endoscopic transsphenoidal surgery, giant pituitary macroadenoma, macroprolactinoma, pituitary adenoma, spontaneous cerebrospinal fluid rhinorrhea, transsphenoidal neurosurgery

## Abstract

Pituitary macroadenomas are neuroendocrine tumors residing in the base of the skull. First-line therapies for prolactin-secreting adenomas (prolactinoma) include medical treatment with dopamine agonists and neurosurgical intervention. Cerebrospinal fluid (CSF) rhinorrhea is a well-known complication following skull base tumor treatment; however, spontaneous CSF rhinorrhea as the initial presenting feature of such tumors is rare. We present two cases of spontaneous CSF rhinorrhea in pre-operative invasive pituitary macroadenomas. Invasive pituitary macroadenomas have the potential to disrupt local skull base structures such as the sellar floor and cavernous sinus. Early warning signs can help aid in prompt diagnosis and successful treatment. Spontaneous CSF rhinorrhea as the initial presenting symptom is rare and may be pivotal in the early diagnosis of pituitary adenomas.

## Introduction

Pituitary adenomas represent 10-15% of all adult intracranial tumors and are the most common type of neuroendocrine tumor of the central nervous system​ [1]. Residing in the central compartment of the skull base in the sella turcica, their unique anatomy gives them a propensity to cause cerebrospinal fluid (CSF) rhinorrhea after surgical intervention. CSF rhinorrhea is a well-known side effect of both medical and surgical therapies for pituitary tumors. Spontaneous CSF rhinorrhea as the earliest presenting feature of pituitary tumors is rare ​[2]. We present two cases of spontaneous CSF rhinorrhea as the initial presenting feature of invasive pituitary macroadenomas. We hypothesize that the skull base invasion by large biologically aggressive pituitary tumors can disrupt the sellar architecture sufficiently to allow CSF leakage prior to any surgical therapy.

## Case presentation

Case 1: Giant pituitary adenoma with adrenocorticotropic hormone (ACTH) expression 

**Figure 1:**
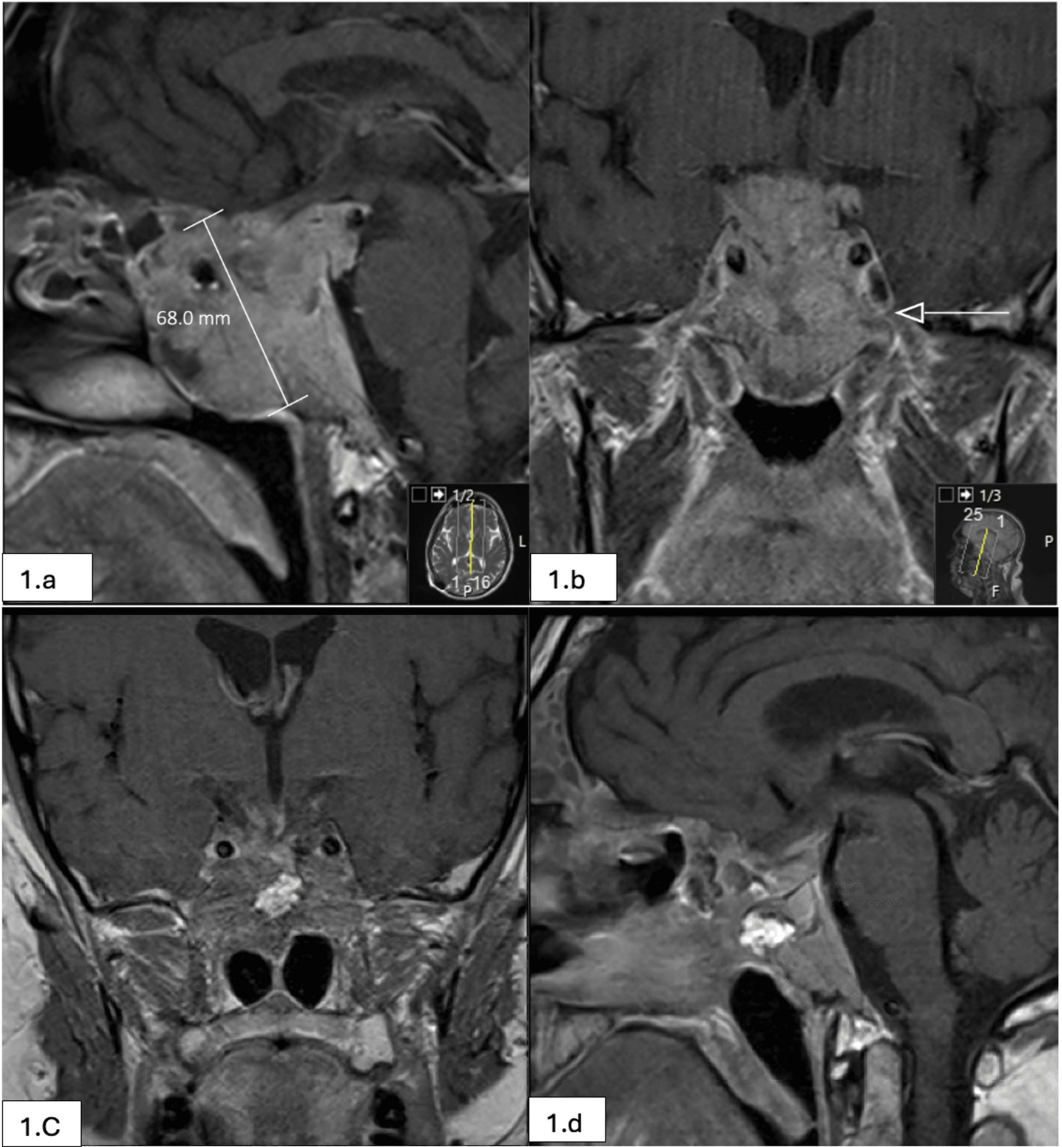
Giant pituitary adenoma with ACTH expression (pre and post-op MRI scans) (a,b): Post-contrast T1 sagittal and coronal images (1.5 Tesla MRI scanner, Siemens Healthcare Laboratory Diagnostics). A large mass lesion occupying the pituitary fossa and chiasmatic cistern and extending inferiorly to replace the sphenoid sinus. The tumor extends into the posterior ethmoid air cells, cavernous sinuses, and into the nasopharynx. Maximum diameter is 68 mm. (c,d): Post-operative MRI scan showing post-contrast T1 sagittal and coronal images (1.5 Tesla MRI scanner). ACTH, adrenocorticotropic hormone

A 54-year-old male presented with a two-month history of headaches, worse when bending forward, and persistent clear liquid discharge from his nose. His symptoms were initially treated as chronic sinusitis by his primary care physician. At this time, he did not have any significant neurological deficit on examination. As the symptoms did not resolve with conservative therapy, further investigations were completed. Cerebral imaging revealed an extensive sellar and parasellar lesion infiltrating the sphenoid sinus, cavernous sinuses, ethmoid air cells, and clivus (Figures 1a-1b). Prior to this, he had no other head trauma, surgery, or other related medical findings that would have indicated a tumor of this size. 

Surgical management was delayed due to a severe COVID-19 infection requiring nursing in the prone position. After some recovery from COVID-19 infection and considering the ongoing high flow CSF leak, he underwent insertion of a programmable ventriculoperitoneal shunt in an attempt to reduce the intracranial pressure and obviate CSF rhinorrhea. This approach was considered due to his condition at the time and to let him fully recover from COVID-19 infection before embarking on a more extensive operation. He had a biopsy of the nasal part of the tumor before his COVID-19 infection, which confirmed the diagnosis of pituitary adenoma. Despite lowering the shunt setting, his CSF rhinorrhea continued. Following a discussion with the skull base multidisciplinary team, it was deemed appropriate to offer him transsphenoidal surgery. He underwent image-guided endoscopic extended transsphenoidal debulking of the sellar and parasallar portions of the tumor. After extradural removal of the tumor, the CSF fistula was identified on the left side of the cavernous sinus junction and adjacent to the carotid artery. This area was sealed with fat graft and tissue glue and reinforced with a nasoseptal flap. Due to the invasion of the cavernous sinus, removal of the intradural portion of the tumor was not attempted. Post-operative hemorrhagic changes were noted in this remnant intradural component of the tumor. The CSF fistula was successfully repaired, and the patient recovered from the operation without complications. Follow-up imaging (Figures 1c-1d) showed a stable residual tumor. He is not on any hormonal replacement at present. 

Pathological analysis showed the tumor to be positive for CAM5.2, an epithelial tumor marker, and synaptophysin, a neuroendocrine tumor marker​ [3]. Ki-67, a classical marker of cell proliferation and aggressiveness, was found to be less than 3% (reference range: >3% is prognostic)​ [4]. While this is a relatively low level of expression for a macroadenoma, it does not necessarily negate its ability to act aggressively toward local structures. The Ki-67 labeling index value relies on lab technique, interpretation, and most importantly, the location of the tissue sample acquired. Tumor cell populations notoriously lack homogeneity, making histological interpretation more challenging​ [5].

Prior to surgery, adrenocorticotropic hormone (ACTH) levels in the patient were found to be 52 ng/L (reference range: <50 ng/L) (Elecsys ACTH Assay, Roche Diagnostics) and prolactin (PRL) levels (ELISA) were 348 mIU/L (normal: 86-324 mIU/L). Subsequent pathological analysis reported a proportion of the adenoma cells positive for ACTH. The absence of endocrine hypersecretion combined with positive ACTH expression indicates a silent corticotroph adenoma. These tumors tend to be clinically more aggressive with local invasion ​[6].

Case 2: Macroprolactinoma 

**Figure 2:**
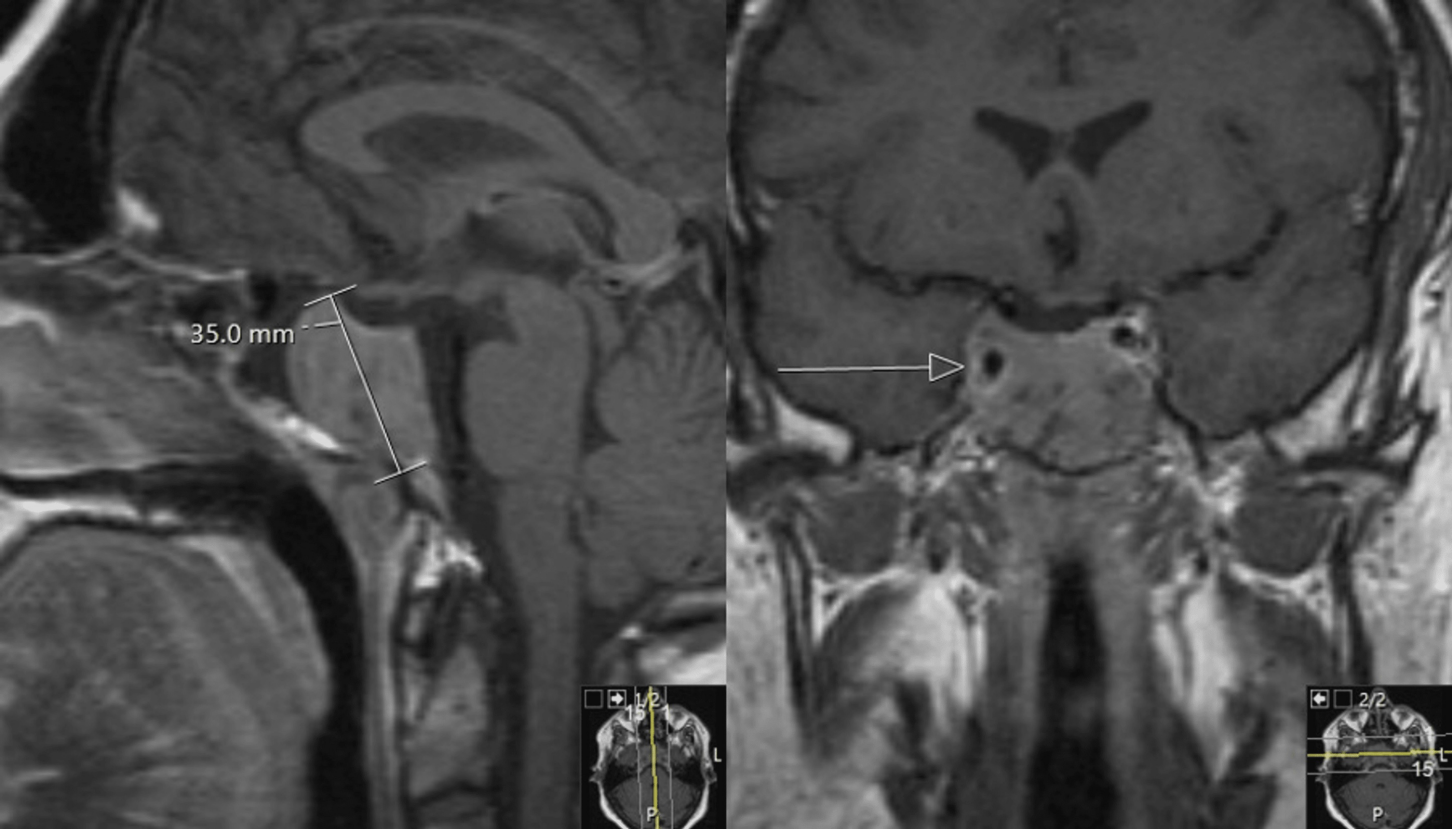
Macroprolactinoma Post-contrast T1 sagittal and coronal images (1.5 Tesla MRI scanner, Siemens Healthcare Laboratory Diagnostics). Giant invasive pituitary lesion replacing the sphenoid sinus and invading the right cavernous sinus (white arrow). Maximum diameter is 35 mm.

A 42-year-old male patient presented to the emergency department with a severe new-onset headache, neck pain, blurry vision, and a two-month history of clear fluid draining from his nose. There was a history of body hair loss and lack of libido prior to this presentation. He presented acutely with neck stiffness and fever in keeping with bacterial meningitis. He was treated for bacterial meningitis, which was followed up with cerebral imaging. MRI revealed an invasive pituitary tumor eroding through the sellar floor (Figure 2). 

The PRL levels in the patient were elevated to 92,000 mIU/L (reference range: <500 mIU/L) [7], indicating that the tumor was functioning as a prolactinoma (ADVIA Centaur Prolactin Assay, Siemens Healthcare Laboratory Diagnostics). Dopamine agonists (DA) are the first-line therapy for prolactinomas. As the dura had already been infiltrated, it was deemed necessary to debulk the tumor and repair the CSF fistula first. The floor of the sella was sealed and bolstered with muscle, and the sphenoid sinus was packed with fat. After recovering from the surgery, the patient was started on medical therapy with hydrocortisone and the DA cabergoline. Upon follow-up one year later, there was no evidence of a recurrence of the CSF leak. DA was stopped after a stable period of 10 years. While there had been no clinical indication of recurrence, imaging follow-ups were complicated by the patient’s claustrophobia and hesitance to have an ordinary MRI. 

## Discussion

CSF rhinorrhea is a known complication of skull base tumors. Open communication between the subarachnoid space and the nasal cavity (fistula) allows for the drainage of CSF out through the nose ​[2]. This abnormal opening is thought to be due to the disruption of the dura mater along with skull base defects [8]. The etiologies of CSF rhinorrhea are vast but tend to be dominated by craniofacial trauma, both physical and iatrogenic ​[9]. A smaller percentage of these cases are deemed spontaneous when there is no clear inciting event [2,10].

Tumors of the pituitary are unique due to their embryonic origin as well as their skull base location​ [11]. Classification and behavior are largely influenced by three main components, namely tumor size, hormone secretion, and local invasion. Each plays a crucial role in the integrity of the dura and the development of a fistula ​[12,13].

Size: Adenomas are defined based on their maximum diameter as microadenomas (<10mm), macroadenomas (>10mm), and giant adenomas (>40mm) ​[12,14]. The cases we presented were of giant (Case 1) and invasive macroadenomas (Case 2). Although the vast majority of pituitary tumors are benign tumors with indolent clinical course, due to their anatomical location, their expansion in size or local invasive behavior may cause compressive effects or CSF fistula as a result of invasion in surrounding bony structures [15].

Hormone secretion: Tumors arising from the pituitary display complex and heterogeneous behavior depending on functional status and hormone involvement [4]. Functioning pituitary tumors possess the ability to secrete one or more pituitary hormones (ACTH, PRL, growth hormone (GH), thyroid-stimulating hormone (TSH), follicle-stimulating hormone (FSH), luteinizing hormone (LH)) [15]. Nonfunctioning tumors do not influence hormone secretion [14]. Case 1 displayed some ACTH expression within the cells of the tumor but did not secrete ACTH. Case 2 displayed an increase in PRL expression at 92,000 mIU/L (reference range: <500 mIU/L) [7]. Hormone secreting tumors may present symptomatically, painting a clinical picture that helps guide clinicians to accurate diagnosis and treatment [16]. Silent pituitary tumors are more insidious and can present with hormone deficiencies or symptoms relating to the damage of local anatomical structures [6,17]. 

Local invasion: Large invasive tumors are more aggressive and involve substantial extrasellar expansion, possessing the potential to disrupt the base of the skull leading to CSF leakage [18]. The breakdown of barriers separating the subarachnoid space and the paranasal sinuses can have a detrimental effect on the brain's blood supply and function [8,19]. Invasive pituitary tumors display gross infiltration of the dura, sphenoid sinus, or skull base [7]. While these types of pituitary tumors are likely to be benign, they tend to be aggressive having an impact on the surrounding anatomical structures [17,20,21]. Clinically aggressive adenomas may exhibit rapid growth, invasion of surrounding tissues, multiple recurrences, and may show resistance to conventional treatments [17]. 

The treatment of pituitary tumors involves a multidisciplinary team with the goal of reducing tumor size, restoring normal hormone levels, and repairing local structures if necessary. This can be achieved through medical therapy and/or surgical intervention [7]. 

The first-line therapy for functioning pituitary adenomas is medical treatment aimed at normalizing hormone levels [12]. Prolactinomas require medical treatment with dopamine agonists [7]. This mainstay in therapy has been shown to normalize PRL levels, re-establish gonadal function, and decrease the size of the tumor by at least 50% [7,22]. While highly successful, the treatment must be monitored closely as subsequent tumor involution may result in the creation of a CSF fistula and rhinorrhea [23-26]. 

For functioning adenomas resistant to medical therapy or non-functioning adenomas, surgical intervention is the treatment of choice ​[24]. Endoscopic transsphenoidal surgery is a common method for resecting tumors of the pituitary [27,28]. Popularized by Harvey W. Cushing in the early 1900s, the sellar region is reached through the nasal cavity, eliminating the need for a more invasive transcranial approach [29]. While the procedure is relatively uncomplicated, it disrupts the meninges and surrounding architecture, allowing CSF to seep out of its proper location [19]. 

CSF fistulas may occur via medical therapy, surgical intervention, or spontaneously. Regardless of the mechanism, it is imperative to repair this abnormal communication. Repairing a nasal CSF fistula is accomplished with endoscopic endonasal closure [28,29]. This procedure uses the patient's own tissue or a biomedical graft to bridge the gap between the intracranial and extracranial spaces [30]. High-resolution CT and MRI imaging are used to help localize the fistula and guide graft placement [31]. 

The diagnosis of CSF rhinorrhea is largely based on clinical presentation but can be supported by endoscopy, radiology, and biomarker technology ​[9,32]. In patients with a history suggestive of CSF rhinorrhea, biochemical markers may aid in early diagnosis [33]. The measurement of β-transferrin can suggest the diagnosis of a CSF fistula prior to visualization through imaging or during surgery [34,35]. For tumors that have already been identified and biopsied, pathological assessment of proliferative potential may help inform treatment and prognosis. Ki-67 index, mitotic count, and p53 positivity have been used widely to reflect tumor proliferation and invasiveness [13,17,36]. 

## Conclusions

In this article, we report two cases of spontaneous CSF rhinorrhea in pre-operative giant pituitary adenomas. The initial symptom of CSF rhinorrhea in large invasive skull base tumors is significant as it is not the traditional presenting symptom​. Non-traumatic, high-pressure CSF leaks have been reported in the literature; however, it is currently unknown to what extent they arise as the initial presenting symptom of giant pituitary adenomas​. Our study highlights the importance of considering unconventional symptoms in the differential diagnosis of pituitary adenomas. We hope our case additions, along with others’ case reports, will help characterize this phenomenon and inform early decision making in skull base surgery procedures. 
